# Simulation-based education model for under-resourced nursing education institutions in Lesotho

**DOI:** 10.4102/hsag.v27i0.1889

**Published:** 2022-10-28

**Authors:** Pule S. Moabi, Ntombifikile G. Mtshali

**Affiliations:** 1 Department of Nursing, Scott College of Nursing, Morija, Lesotho; 2 College of Health Sciences, School of Nursing and Public Health, University of KwaZulu-Natal, Durban, South Africa

**Keywords:** simulation-based education, nurse educator, student, model, level

## Abstract

**Background:**

The changing landscape of nursing education to competency-based education has strengthened the importance of simulation learning in the process of developing the required graduate competencies.

**Aim:**

This study aimed to develop a model that guides the implementation of simulation-based education (SBE) in under-resourced nursing education institutions in Lesotho.

**Setting:**

Four Nursing Education Institutions in Lesotho.

**Methods:**

An explanatory sequential mixed methods design was adopted. Sampling methods included stratified systematic random, purposive and systematic sampling. The total sample was 390 comprising students, nurse educators and principals. Data were collected through questionnaires, focus group discussions and in-depth unstructured individual interviews. Statistical analysis was employed for quantitative data while a grounded theory approach guided the qualitative data analysis and model development.

**Results:**

Implementation of simulation emerged as a multilevel, multi-actor and multistage process of adopting, introducing and implementing SBE. This education takes place in a simulated environment that serves as a connecting bridge between the learning of theory in the classroom and clinical learning in real-life settings. The model generated from this study has simulation implementation as the main concept that is supported by four major concepts: (1) simulation initiation at the strategic level, (2) simulation implementation at the tactical level, (3) simulation implementation at the operational level and (4) simulation outcomes.

**Conclusion:**

Successful implementation of simulation requires buy-in from key stakeholders. Simulation-based education policy, competent facilitators and a well-resourced clinical skills laboratory may facilitate the development of the required competencies.

**Contribution:**

The study provides guidance on how SBE can be implemented in resource-limited settings.

## Introduction

Simulation-based education (SBE) is defined as an educational process where real clinical practice is replicated in a safe environment with the aim of enhancing students’ confidence and competence (Unver et al. 2018). In SBE, nursing students learn through imitation of real patient cases and scenarios, as scenarios are used to stimulate critical thinking and decision-making (Krishnan, Keloth & Ubedulla 2017).

There are several models that have been developed to provide direction to simulated learning in nursing education, and simulation model use is influenced by the availability of simulation resources, simulators, simulation appreciation by various stakeholders, its pros and cons and experience of the users. Simulation models differ in terms of the planning of the simulation experience, implementing the simulation scenario and evaluating the simulation experience. These models include the World Health Organization (WHO) simulation model, the practice-based simulation model, the collaborative clinical simulation model and Jeffries’ simulation model (Guinez-Molinos et al. 2017:197; Jeffries 2005; Martins et al. 2018; Park et al. 2013).

In the WHO simulation model, when planning for simulation experience, the facilitator must create scenarios and prepare the environment to be used. The script of the scenario must include the learning objectives, a full description of the scenario, participants’ roles and resources needed for the scenario (Martins et al. 2018). When implementing the simulation scenario, it will run in three steps according to Martins et al. (2018), and these steps are briefing, action and debriefing. During evaluation of the simulation experience, the simulation process assessment is conducted. The simulation process assessment focuses on whether the simulation programme was executed according to the plan and challenges experienced. This kind of assessment, according to Martins et al. (2018), will help the facilitator when planning for the future simulations.

One model that plans for the simulation experience and implements simulation scenarios in a similar manner to the WHO simulation model is the practice-based model (Park et al. 2013). During the planning of the simulation in the practice-based model, cases are designed for students to manage. Cases can be in the form of patient case or scenarios showing various people or situations (Park et al. 2013). When implementing a scenario, the learners take an active role as a nurse or a clinician in a simulated environment. During the simulation round, learners are given 10–15 min for prebriefing, 10–15 min for the simulation exercise and 20–30 min for debriefing. When evaluating the simulation experience, assessment focuses on multiple methods of assessment that cover knowledge, skills, attitudes, critical thinking and decision-making (Park et al. 2013).

A collaborative clinical simulation model plans simulation experiences differently from the aforementioned models (Guinez-Molinos et al. 2017). When planning for simulation experiences in this model, students are divided into three small groups, and group members range from three to five. The groups are then seated in different rooms and design a short clinical case based on the differential diagnosis given by the facilitator (Guinez-Molinos et al. 2017). When implementing the scenario, one group applies a case scenario designed by them to the other group, and the other group performs the assigned scenario, while the third group is observing the simulation in a separate room. During evaluation of the simulation experience, a guided, planned and well-structured reflection on the simulated activity is conducted after each cycle of simulation. The facilitator and the observational group will provide comments on the performance, and the reflections will be centred on what went well, called ‘pluses’, and what students would like to change about their performance, called ‘deltas’ (Guinez-Molinos et al. 2017).

When planning for a simulation experience in Jeffries’ simulation model, the facilitator needs to consider the following: objectives, planning, fidelity, complexity, cues and debriefing. Objectives need to be clearly written, and these objectives should match students’ knowledge and experience (Jeffries et al. 2015). During implementation of the simulation scenario, students need to be provided with cues as they progress during simulation activity, and when evaluating simulation experience, skill performance is evaluated by means of checklists, and remedial teaching is provided to students in need. Students are also offered the opportunity to evaluate their simulation experience, and the evaluations are in the form of questionnaires about students’ experiences (Jeffries 2005).

The given models do not apply to SBE implementation in resource-limited settings. Simulation-based education was first introduced in Lesotho in 2012 by one of the developmental partners, the Nursing Education Partnership Initiative (NEPI). Implementation of simulation is facing various challenges, such as limited human resources to staff the simulation laboratories, limited simulation equipment and unsuitable simulation implementation (Munangatire & Naidoo 2017; NEPI 2012:6). Therefore, this study aimed to develop a model that guides the implementation of SBE in under-resourced nursing education institutions (NEI) in Lesotho.

## Research methods and design

The process of developing the model was carried out in two phases, namely empirical and model development phases.

### Study design

**FIGURE 1:**
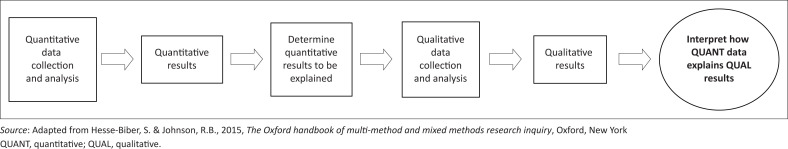
Explanatory sequential mixed methods design.

An explanatory sequential mixed methods design was adopted in this study, and a grounded theory approach was used to guide the model development process. The researchers utilised an explanatory sequential mixed methods design because they wanted to collect and analyse quantitative data from the nurse educators and students to inform the types of questions to be asked to participants during interviews when probing. For the quantitative part, the following were research questions that guided the study: (1) what is the state of readiness of NEIs in Lesotho to implement SBE? (2) What are student nurses’ perspectives on SBE in Lesotho? The qualitative aspect was guided by the following research questions: (1) how is simulation based-education currently being implemented by NEIs in Lesotho? (2) How can simulation based-education be implemented effectively in resource-constrained NEIs? Figure 1 shows the explanatory sequential mixed methods design used, as adapted from Hesse-Biber and Johnson (2015).

The model emerged from the integrated findings of the quantitative and qualitative data. According to Corbin and Straus (2015), a model in a grounded theory approach is generated through systematic, interactive and rigorous data collection and a constant comparative analysis process to ensure emergence of the model from the data.

#### Research setting

Four NEIs that are under the Christian Health Association of Lesotho (CHAL) were research sites. These four NEIs are privately owned by various churches; they have similar programmes, and their teaching and learning is guided by a competency-based curriculum. The public NEIs were not included because their teaching and learning are still guided by a content-based curriculum; they write separate examinations, as opposed to CHAL institutions, and they have more government financial support than CHAL institutions.

#### Sampling

The study population comprised the nurse educators (NEs), the principal nurse educators (PNEs) and students from the four CHAL NEIs in Lesotho. A total of 18 NEs were from NEI 1, 15 were from NEI 2, 15 were from NEI 3 and the other 15 were from NEI 4. For the quantitative aspect, the total population sampling method was employed because the population of the NEIs was manageable. For nursing students, the population comprised second-year, third-year and midwifery students from the four selected NEIs. The first-year students were excluded because the institutions did not have first-year intake because of coronavirus disease 2019 (COVID-19) restrictions. The researchers used stratified systematic random sampling, with each level of student forming a stratum. Sample proportions were identified per institution, and from the proportions, the researchers identified the sample size using the Qualtrics sample size calculator (SAP SE, Walldorf, Germany) with the confidence level of 95% and margin of error of 5%. The recommended sample was selected from the student population, and table of random numbers were used to select every third student. Table 1 shows the quantitative sample of NEs while Table 2 shows the quantitative sample of students.

**TABLE 1:** Sample size for educators from the findings of the study.

Institution	Population	Sample size
NEI 1	20	18
NEI 2	16	15
NEI 3	17	15
NEI 4	16	15

**Total**	**69**	**63**

NEI, nursing education institution.

**TABLE 2:** Sample size for students from the findings of the study.

Institution	Sample size	Sample proportion per level of study %	Sample per level of study
NEI 1	36	Second: 39.5	Second: 14
		Third: 25	Third: 9
		Midwives: 35.5	Midwives: 13
NEI 2	64	Second: 29.3	Second: 19
		Third: 27.4	Third: 17
		Midwives: 43.3	Midwives: 27
NEI 3	76	Second: 32.7	Second: 25
		Third: 36.7	Third: 28
		Midwives: 30.6	Midwives: 23
NEI 4	99	Second: 24.6	Second: 25
		Third: 40.7	Third: 40
		Midwives: 34.7	Midwives: 34

**Total**	**275**		**275**

NEI, nursing education institution.

In the qualitative phase, the population was made up of 28 nurse educators, 8 second year students, 8 third year students and 8 midwifery students, who were purposely selected because they have been exposed to SBE. Theoretical sampling was used when emerging categories needed to be refined until data saturation was reached as no new information emerged (Corbin & Straus 2015). Table 3 and Table 4 show qualitative samples of educators and students, respectively.

**TABLE 3:** Sample size for educators from the findings of the study.

Institution	Population	Sample size
NEI 1	20	7
NEI 2	16	7
NEI 3	17	7
NEI 4	16	7

**Total**	**69**	**28**

NEI, nursing education institution.

**TABLE 4:** Sample size for students from the findings of the study.

Institution	Population size	Sample size
NEI 1	76	6
NEI 2	201	6
NEI 3	98	6
NEI 4	118	6

**Total**	**493**	**24**

NEI, nursing education institution.

#### Data collection

As the design used in this study was explanatory sequential mixed methods, the researchers first collected the quantitative data and then the qualitative data. The following was carried out for the quantitative data collection: As a result COVID-19 restrictions during collection of quantitative data, the questionnaires were administered online. For educators, the researchers adopted a survey tool developed by Foisy-Doll and Leighton (2017), which is used to assess the organisational readiness for SBE. The adopted questionnaire had 25 questions, and the responses were rated on a five-point Likert scale. This five-point Likert scale had the following options: none at all (1), a little (2), somewhat (3), moderately (4) and very much (5). In relation to the students’ questionnaire, the researchers adopted questions from some of the tools used to evaluate simulated learning, and such tools included: the National League for Nursing instrument that assess simulation design (National League for Nursing 2005), a survey instrument from the National League for Nursing that assesses the educational practices and a survey tool used by Leighton, Mudra and Macintosh, which evaluated the psychometric properties of the modified Simulation Effectiveness Tool (SET). The questionnaire had 66 questions, and the responses were rated on a six-point Likert scale. This six-point Likert scale has options where participants chose one option per question: strongly disagree with the statement (1), disagree with the statement (2), undecided (3), agree with the statement (4), strongly agree with the statement (5) and not applicable (6).

For qualitative data, focus group discussions with the aid of an interview guide (which was based on the collected and analysed quantitative data) were used to collect data from PNEs, NEs and students. Four FGDs were conducted for both NEs and students, culminating in a total of eight FGDs, and each FGD comprised six participants. Each FGD lasted from 45 min to 90 min. For the PNEs, in-depth face-to-face unstructured individual interviews and field notes were used to collect data, and the interviews were recorded with a voice recorder, guided by the central questions. The following central questions guided the FGDs with the nursing students:

1. Please tell me about your experiences with simulated learning?
2. How could your experience be improved?
3. What do you understand is the purpose of simulated learning in nursing education?

For the PNEs and the NEs, the following central questions guided the interviews:

1. How do you currently conduct simulated learning in your institution?
2. Tell me about your experiences as the PNE ‘or’ an educator in relation to simulated learning?
3. How can simulated learning be improved?
4. Please share with me your understanding of simulated learning in nursing education?

Facilitative communication skills assisted the researchers to ensure that the research questions were adequately addressed (Mavundla, Poggenpoel & Gmeiner 2001). The interviews were conducted from 25 August 2020 to 05 January 2021.

#### Data analysis

For quantitative aspect of the study, the Statistical Package for the Social Sciences (SPSS) version 27 (IBM Corporation, Armonk, New York, United States) was used to code data for analysis. Descriptive statistics were used to analyse data. Qualitative data collection and analysis were conducted concurrently as the researchers were utilising grounded theory (Corbin & Strauss 2015). The recordings from the audio recorder were transcribed verbatim, and line-by-line analysis was conducted by reading the transcripts line-by-line as part of open coding. Open coding yielded categorisation of similar codes as constant comparison of data were performed. This is where the researchers moved back and forth when identifying similarities among and differences between emerging categories (Corbin & Strauss 2015; Tie et al. 2019). The categorised codes were refined by conducting axial coding (Corbin & Strauss 2015; Tie et al. 2019).

While conducting the qualitative data analysis, emerging categories were revisited in order to identify similarities and differences between and among them (Corbin & Strauss 2015; Tie et al. 2019). Emerging categories were organised into categories and subcategories according to the six elements in Strauss and Corbin’s data analysis model (Charmaz 2006; Corbin & Strauss 2015). The researcher then refined the emerging categories into an analytic model through the process of selective coding (Corbin & Straus 2015). During the selective coding, the core category that emerged from reduction and constant comparison was SBE implementation. The quantitative and the qualitative findings were then integrated. Narrative integration was performed by describing the quantitative and qualitative research findings in a single report, as presented in a separate article (Fetters et al. 2013; McCrudden & McTigue 2019; Moseholm & Fetters 2017). The weaving approach to narrative integration was conducted by writing both quantitative and qualitative findings together on a concept-by-concept basis.

The core category became the centre of attention for analysis, and it guided further theoretical sampling and data collection (Corbin & Straus 2015). The core category was then related to other categories through explicating a story from the interconnection of the categories and also validated by conducting a focused literature search (Corbin & Straus 2015). Relationship specification was conducted by re-examining the influence that exists between the concepts and noting the relationships down in a diagrammatic form. Then the model was illustrated in a diagrammatic form to see how and why the concepts are related to each other (McCrudden & McTigue 2019).

#### Scientific rigour

Items adopted from questionnaires that have been tested and found valid ensured content validity, and external validity was ensured by including a representative sample of the population (Creswell & Creswell 2018; Gray, Grove & Sutherland 2017). The reliability coefficient of the students’ data collection tool was 0.88, while for the educators’ tool it was 0.84, and those are the acceptable values as the range is from 0.8 to 0.9 (Gray et al. 2017). In order to ensure trustworthiness of the findings, Lincoln and Guba’s (1985) criteria were utilised. Credibility was ensured by prolonged engagement with the participants, and field notes were taken. Dependability was ensured by reporting the processes and study methods in detail, while confirmability was guaranteed by availing audio-recorded information of raw data and field notes to an external audit. This is where an external person assessed the data analysis process, which was employed by the researchers. Thick description of the phenomena under investigation also ensured transferability (Creswell & Creswell 2018). Regarding researchers’ positionality, when collecting data in an institution at which the first author is employed, a research assistant who is not an employee of the institution was recruited to assist with data collection. The second author is a professor in a university and does not have an affiliation with any of the data collection sites.

#### Ethical considerations

The study was ethically approved by University of KwaZulu-Natal (ethical clearance number: HSSREC/00001411/2020) and the Ministry of Health Lesotho (ethical clearance number: 88-2020). Heads of the NEIs provided permission for the study to be conducted. Full information relating to the study was provided to the participants. They were informed that participation in the study is voluntary and they can withdraw from the study at any time (Creswell & Creswell 2018).

## Results and discussion

The article only presents the model, while other findings (qualitative and quantitative) have been published in other academic journals (Moabi & Mtshali 2021, 2022). Through refinement, a model that guides implementation of SBE in under-resourced NEIs in Lesotho was illuminated through the process of storyline (Tie et al. 2019).

### Model development phase

Seven elements of model development according to Chinn and Kramer (2011) are used to present the model on the implementation of SBE in under-resourced NEIs in Lesotho that emerged from this study. These elements include: (1) the purpose of the model, (2) identification of the concepts of the model, (3) assumptions of the model, (4) definition of concepts of the model, (5) nature of relationships between and among concepts and (6) stating the structure of the model.

### Purpose of the model

The purpose of the model is to: (1) serve as a framework for under-resourced NEIs to fully implement SBE, (2) provide guidance to the managers and administrators on how to support SBE implementation, (3) provide guidance to the nurse educators in under-resourced nursing education on how to facilitate simulation-based learning using the available resources and (4) guide NEIs in evaluating the implementation of SBE in their institutions.

#### Basic assumptions

The simulation-based education model for under-resourced nursing education institutions in Lesotho has been purely created based on assumptions of the study results. The assumptions are: (1) SBE implementation is cyclic, (2) administrative and managerial support are crucial in SBE implementation, (3) simulation-based teaching requires detailed planning and preparation and (4) student support during simulation is essential.

#### Concepts and definitions

During the model development, SBE implementation was identified as the main concept, with four major concepts attached to it. There are four major concepts directly linked to the main concept: (1) SBE initiation at strategic level, (2) SBE implementation at tactical level, (3) SBE implementation at operational level and (4) SBE outcomes. Attached to the major concepts are subconcepts referred to as second-level concepts. The subconcepts are presented later under the definition of each major concept.

The core concept of SBE implementation in this model is defined as a multilevel, multi-actor and multistage process of adopting, introducing and implementing SBE. This education is implemented in a simulated learning environment that serves as a connecting bridge between learning theory in the classroom and clinical learning in real-life settings. This is congruent with what Unver et al. (2018) and Krishnan et al. (2017) suggest as they explain that simulation is a strategy that is tailored to foster development of decision-making skills and critical thinking in students in a safe environment, and set of conditions are created artificially in order to study something that is possible in real life. Implementing SBE is regarded as multilevel with multiple actors because implementation encompasses three levels: strategic, tactical and operational levels where multi-actors are involved. At the strategic level, actors include leadership and management; at the tactical level and operational level, actors are nurse educators, administrators, support staff and students, with leadership and management providing guidance and support as required.

*Simulation-based education initiation at the strategic level*: The concept ‘SBE initiation at the strategic level’ refers to the stage of embarking on the adoption of SBE in line with the country’s agenda to transform nursing education. This takes place at the top or administrative level in the institution. It is at this stage where a shared vision and commitment is developed as well, as policy frameworks and resources are made available to support the change. This main concept of SBE initiation at strategic level includes five subconcepts: (1) shared SBE vision, (2) SBE policy, (3) funding for SBE policy implementation, (4) external change facilitator and (5) SBE champions.

Shared vision refers to the presence of clear and well-communicated messages between management and the faculty on the future of SBE where all parties involved in the SBE initiation have a clear understanding of the importance of SBE. To create shared vision, the management of the institutions must allow simulation facilitators and faculty to take part while developing or revising the institutional strategic plans. On the other hand, simulation policy refers to a legal document that directs the action of leaders of institutions, faculty, students and various stakeholders involved in SBE. In this study, simulation policy must have the following aspects: simulation lab operating hours, attendance and booking, acceptable behaviour, responsibilities, physical safety, stock and equipment control, maintenance and care of simulators, which is in line with what Ross (2012) recommends. As low-resource settings, the NEIs must utilise the existing capacity within their institutions (simulation facilitators and faculty) to develop simulation policies and must not source an external consultant, as this can be an expensive exercise.

The external change facilitator is an external expert who acts as a change agent within the institution with the responsibility to facilitate and monitor the implementation of SBE by the nursing education institution. The external change agent has to ensure that adequate capacity is built within the institution by the time the change facilitators leave the institution, as stated in Uys and Gwele (2005). In this study, the external change facilitator was sponsored by various developmental partners. To ensure sustainability of the innovation, the external change facilitator must assist the institutions to develop SBE sustainability plans. In the context of this study, as the NEIs are low-resource settings, the institutions must forge strategic partnerships with universities or colleges who are leaders in SBE implementation. This will assist the institutions to acquire simulation experts from the universities or colleges that will act as change facilitators.

Funding for SBE policy implementation is considered as ways in which the NEIs budget, mobilise and acquire funds that are necessary to implement SBE policies in the institutions. Effective policy implementation is dependent upon the availability of funding to support its implementation (Mugwagwa, Edwards & De Haan 2015:1). In this study, SBE implementation can be funded in part through institutional budgets, but major funding can be from the developmental partners and from institutions’ income generating projects as the institutions are resource limited (Michaels-Strasser et al. 2018; Middleton et al. 2014; NEPI 2016). Institutional budgets and annual operational plans need to include simulation, and the budget allocation should be informed by cost analysis reports. Nursing education institutions in the context of this study can source funding to support SBE implementation by writing proposals to potential funders. Simulation-based education champions in this model are the simulation facilitators or faculty members who are advocates of SBE and are knowledgeable, committed, passionate and able to influence other colleagues to adopt simulation (Foisy-Doll & Leighton 2017). In this study, SBE champions are nurse educators who have been trained on SBE and are currently facilitating clinical teaching.

*Simulation-based education implementation at the tactical level*: The SBE implementation at the tactical level refers to implementation at the mid-level where substantial and detailed planning and preparation takes place. The level where structures, processes and tools are developed for effective implementation of SBE at operational level. Activities at this level are guided by decisions and framework from the strategic level. Simulation-based education implementation at tactical level has three subconcepts: (1) teaching and learning infrastructure development, (2) curriculum and materials development and (3) human resources mobilisation and capacity building.

Teaching and learning infrastructure development refers to the erection of the simulation laboratory, creating an immersive simulation environment, commissioning the newly built infrastructure and procuring adequate simulation equipment (manikins) and supplies. According to Benckendorff et al. (2016), institutions can still implement SBE in the absence of high-fidelity manikins, and they can opt for low-fidelity simulation but pitch the simulation scenarios to a higher level that will stimulate critical thinking and problem solving in the students. As resource-limited settings, the findings of this study show that an immersive simulation environment in the NEIs in Lesotho has been partially created, as the laboratories have three simulation rooms, a reflection room, storeroom and toilets. There is adequate water supply and lightning and electrical outlets in each room, while oxygen used is stored in cylinders and the suctioning systems used are electrical suctioning machines. In the context of this study, because of the fact that the institutions may have financial challenges in procuring high-fidelity manikins, the low-fidelity manikins, which are affordable, can be procured; thus, simulation scenarios can be pitched at a higher cognitive level.

One of the subconcepts linked to the activities at the tactical level is SBE curriculum and materials development, where the faculty in collaboration with the simulation facilitators develop SBE curriculum content to be used, explore ways in which SBE can be integrated into the curriculum and also develop materials and tools to be used. According to Riabtseva, Reynolds and Gisin (2015), tools and materials should include development of simulation scenarios, simulation lesson plans, training agenda and assessment and evaluation tools, which include checklists and logbooks. As the institutions are resource limited and they cannot afford to pay for services of a consultant to assist on SBE integration into the curriculum and materials development, the NEIs can utilise their already existing external change facilitators as they are experts in simulation.

In the context of this model, human resources refer to qualified nurse educators who conduct teaching and learning in simulation and are also referred to as clinical skills laboratory coordinators ‘or’ simulation facilitators. In this study, it is recommended that facilitator–student ratio should be 1:10 as a resource-limited setting, but currently the ratio is 1:30 in almost all the NEIs. The ratio recommended by Krishnan et al (2017) of 1:3 is challenging for resource-constrained settings. The simulation laboratories are staffed by simulation facilitators and there are neither administrators nor technicians in the simulation laboratories. To ensure that clinical practice is up-to-date with the current developments, preceptors who are trained nurses who mentor students in clinical areas need to be incorporated during simulation (Riabtseva et al. 2015). The facilitators and preceptors need to be trained on how to facilitate teaching in learning in SBE (Riabtseva et al. 2015). Nursing education institutions are transitioning from the content-based curriculum to the competency-based, which is learner centred. This implies that there will be less workload on the side of the educators, and the educators who have less workload can be redeployed to the simulation laboratory to add to the already existing simulation team (Griewatz, Simon & Lammerding-Koeppel 2017).

*Simulation-based education at the operational level*: The major concept of SBE at the operational level refers to a process where plans and vision are translated into reality, and it involves two stakeholders, which are teachers and students and each has a role to play. This is where simulation teaching actually occurs. This major concept is supported by three subconcepts which are: (1) multiphase teaching and learning process, (2) the nature of the teacher and (3) the nature of the learner.

The subconcept ‘multiphase teaching and learning’ refers to six cyclical phases that students need to pass through during simulation learning. Based on the assumption that learning is a transformative process, the multiphase process is important as learners grow from one stage to the next as described by Kolb (2014). In this model, the phases are: (1) concrete experience, (2) reflective observation, (3) abstract conceptualisation, (4) active experience, (5) assessment of learning (formative and summative) and (6) evaluation of SBE.

In this model, concrete experience can be demonstrated when students are involved in carrying out various skills in the simulation laboratory. Students must be offered a chance to be actively involved in carrying out nursing procedures on the manikins and the standardised patients in the simulation laboratory. Reflective observation can be demonstrated when the simulation facilitators provide a chance to the students to reflect on their simulation experience, their observations and feelings about the experience. To ensure abstract conceptualisation, after the debriefing process, the students must critically reflect on their simulation learning experiences and develop their own ideas. This is where they will explain that after the simulation experience, they are able to identify what can cause complications when performing nursing skills, such as intramuscular injection.

Active experience in this model can be ensured by creating various scenarios, and students must be given the opportunity to manage their scenarios using their own decisions. Formative and summative clinical assessment can be conducted via Objective Structured Clinical Examinations (OSCEs). Various stations can be set and manned by simulation facilitators, nurse educators and preceptors. To evaluate SBE, after a simulation round, students must be taken into the reflection room in order to evaluate their simulation experience. Students must be given chance to describe the aspects they liked during that round of simulation, the challenges they met and how future simulations can be improved.

In this model, during the multiphase teaching and learning, students in the simulation laboratory will move from the level where they are less competent to conduct a skill to a level where they are competent to conduct a nursing skill. This is termed zone of proximal development (ZPD) as students work in collaboration with a skilled facilitator in constructing knowledge and skill (Fani & Ghaemi 2011). According to Shabani, Khatib and Ebadi (2010), through scaffolding, students are assisted to become independent and competent, and this is achieved by assisting students to complete small, manageable steps in order to reach the desired goal. During simulation teaching, students will pass through the four stages of the ZPD (assisted performance, unassisted performance, full internalisation and de-atomisation) as described by Ahmed (2017) and Fani and Ghaemi (2011). If the NEIs with low resources have external change facilitators, simulation champions and trained personnel, students will be able to pass the four stages of the ZPD through assistance of a competent faculty.

The subconcept ‘the nature of the teacher’ refers to a qualified nurse educator who facilitates SBE and has various qualities such as being a facilitator, being innovative, using evidence-based teaching, understanding student diversity, providing instructional support and having mutual respect. This subconcept is important because the teacher needs to understand the characteristics of learners, including class size, diversity, disciplinary background, level and prior training, as described by Benckendorff et al. (2016); the teacher must also facilitate instructional support while utilising evidence-based teaching (Lerang et al. 2021). In this model, the simulation facilitators must have facilitation skills and must not have full control over students’ learning by not allowing students to have maximum control over their learning. For the simulation facilitators to be competent, training on SBE facilitation is required and institutions may have challenges for paying for training of the simulation facilitators. The low-resourced NEIs can utilise their existing partnerships with partner universities or colleges to provide affordable SBE training to their faculty members.

The nature of the learner as the subconcept that constitutes the operational level refers to nursing or midwifery students with diverse characteristics. The learner is viewed as an adult, active learner, who is self-directed with high expectations and is practical and outcome focused. Billings and Halstead (2009) explain that adult learners have different learning styles, and it is the responsibility of an educator to create various activities that accommodate different learning styles. Simulation should always be hands-on as a form of experiential learning. Through hands-on practice, students will develop competence in nursing skills by practising them in real or simulated areas (Taylor & Hamdy 2013). In this model, students must be treated as adult learners and be given open access to the simulation laboratory so that they can practise in their own time, and this will allow them to have hands-on experience.

*Simulation-based education outcomes*: The major concept ‘outcome’ refers to desired perceived ‘or’ actual benefits of implementing SBE to the students, NEIs and the healthcare system. Simulation was adopted in Lesotho because almost all the nursing training institutions had inadequately equipped demonstration rooms. This was exacerbated by poor supervision of nursing students during clinical placement (NEPI 2012). The benefit of SBE to the students as a subconcept is defined as the advantage that students receive when they are involved in simulation. In this model, the simulation outcome is improvement of the students’ confidence and competence to carry out nursing procedures. Martins et al. (2018) contend that confidence and competence are enhanced by repeated simulation experiences.

The benefit of SBE to the NEIs as a subconcept refers to the advantage gained by NEIs with limited resources who utilise SBE. The data of the study show that the NEIs can benefit from the implementation of SBE, as there are limited clinical sites and simulation offers an opportunity for clinical placement. According to Radford (2018), NEIs have a shortage of clinical sites because they are competing for such sites, and simulation can play a vital role in assisting to bridge theory and practice gap.

The benefit of SBE to the healthcare system as a subconcept of outcomes is defined as the gain that the healthcare system receives when SBE is implemented by the NEIs. The study’s findings indicate that patient safety can be enhanced via simulation because students practise in a safe environment before transiting to real patients. Liaw et al. (2014) explain that in simulation, invasive nursing skills can be learned and practised by the students on manikins.

#### Relationships between concepts and the structure of the model

**FIGURE 2:**
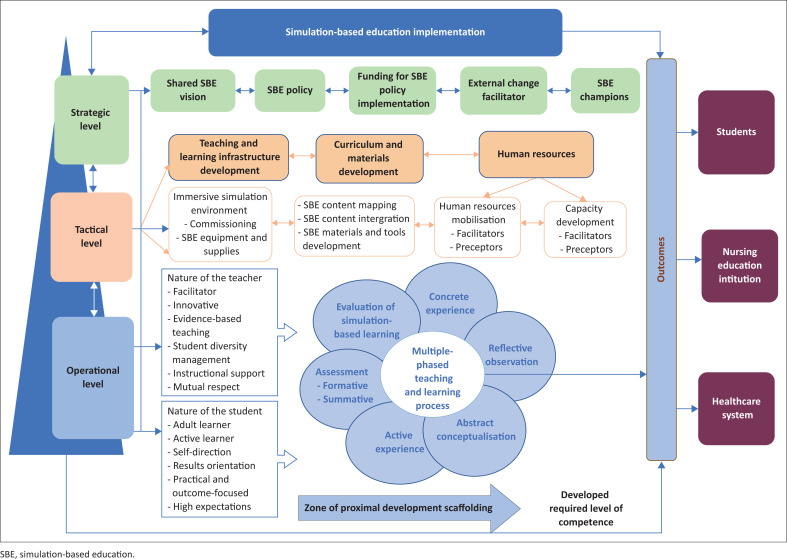
A simulation-based education model for under-resourced nursing education institutions in Lesotho from the study findings.

According to Walker and Avant (2014), concepts are the basic building blocks of a model and they comprise mental image of a phenomenon or an idea. Stating relationships between concepts shows a link among concepts (Chinn & Kramer 2011). In the model that guides the implementation of SBE, the researchers used arrows to provide links among and between the concepts. Simulation-based education implementation is the main concept in this model. As indicated by the arrows, SBE implementation is linked to four major concepts, which are (1) initiation at the strategic level, (2) implementation at the tactical level, (3) implementation at the operational level and (4) SBE outcomes. Figure 2 shows the simulation-based education model for under-resourced nursing education institutions in Lesotho.

### Recommendations

Existing capacity in the NEIs must be utilised to develop simulation polices, and the institutions must forge strategic partnerships with universities or colleges who are leaders in SBE implementation, as this will assist the NEIs to acquire affordable technical assistance and SBE trainings. As the NEIs are resource constrained, they must source funds to support SBE implementation from various potential developmental partners. The low-resource settings must take account that simulation does not only involve use of the high-fidelity manikins, but low-fidelity manikins can be utilised and scenarios can be pitched at a high cognitive level. Human resources are a challenge in SBE implementation, but faculty members with less workload can be redeployed into the simulation laboratory. As a result of challenges of time scheduling for simulation, students need to be given open access to the simulation laboratory, and this is where students can practise on their own time.

### Strengths and limitations

This study utilised a mixed methods design, which assisted in reduction of the threat to the validity of the findings because the strengths of one method compensated for the weaknesses of the other method during data collection (Hesse-Bier & Johnson 2015). This is a novel study as a model to guide implementation of SBE, considering that other aspects such as resource availability were developed. The model is unique because it provides guidance on how the management of the NEIs can support SBE implementation considering the fact that the institutions are resource-constrained.

The following are considered to be the limitations of this study: the study cannot be generalised to all settings as it is restricted to low-resource settings. First-year students were not part of the study because the institutions did not have first-year intake because of COVID-19 restrictions during data collection. As a result of the fact that quantitative data were collected using online surveys, there is a likelihood that some participants may have answered the survey more than once.

## Conclusion

The WHO (2010) advocates for the strengthening of nursing and midwifery education utilising various approaches, such as adoption of curricula to suit population needs and utilisation of simulation as one of the pedagogies. The newly developed model, described as a multilevel, multi-actor and multistage process of adopting, introducing and implementing SBE, can play a vital role in ensuring that nursing and midwifery education is strengthened. Champions from the institutions are the appropriate individuals to implement the model in their respective institutions. Nursing education institutions with similar contexts to Lesotho can adopt or adapt the whole model or parts of the model when implementing SBE.
